# Computational modeling allows unsupervised classification of epileptic brain states across species

**DOI:** 10.1038/s41598-023-39867-z

**Published:** 2023-08-18

**Authors:** Isa Dallmer-Zerbe, Nikola Jajcay, Jan Chvojka, Radek Janca, Petr Jezdik, Pavel Krsek, Petr Marusic, Premysl Jiruska, Jaroslav Hlinka

**Affiliations:** 1 Department of Complex Systems, Institute of Computer Science, Czech Academy of Sciences, 182 00 Prague, Czech Republic; 2 Department of Physiology, Second Faculty of Medicine, Charles University, 150 06 Prague, Czech Republic; 3 National Institute of Mental Health, 250 67 Klecany, Czech Republic; 4 Department of Circuit Theory, Faculty of Electrical Engineering, Czech Technical University in Prague, 166 27 Prague, Czech Republic; 5 Department of Paediatric Neurology, Second Faculty of Medicine, Motol University Hospital, Charles University, 150 06 Prague, Czech Republic; 6 Department of Neurology, Second Faculty of Medicine, Motol University Hospital, Charles University, 150 06 Prague, Czech Republic

**Keywords:** Biophysical models, Biophysical models, Inhibition-excitation balance

## Abstract

Current advances in epilepsy treatment aim to personalize and responsively adjust treatment parameters to overcome patient heterogeneity in treatment efficiency. For tailoring treatment to the individual and the current brain state, tools are required that help to identify the patient- and time-point-specific parameters of epilepsy. Computational modeling has long proven its utility in gaining mechanistic insight. Recently, the technique has been introduced as a diagnostic tool to predict individual treatment outcomes. In this article, the Wendling model, an established computational model of epilepsy dynamics, is used to automatically classify epileptic brain states in intracranial EEG from patients (n = 4) and local field potential recordings from in vitro rat data (high-potassium model of epilepsy, n = 3). Five-second signal segments are classified to four types of brain state in epilepsy (interictal, preonset, onset, ictal) by comparing a vector of signal features for each data segment to four prototypical feature vectors obtained by Wendling model simulations. The classification result is validated against expert visual assessment. Model-driven brain state classification achieved a classification performance significantly above chance level (mean sensitivity 0.99 on model data, 0.77 on rat data, 0.56 on human data in a four-way classification task). Model-driven prototypes showed similarity with data-driven prototypes, which we obtained from real data for rats and humans. Our results indicate similar electrophysiological patterns of epileptic states in the human brain and the animal model that are well-reproduced by the computational model, and captured by a key set of signal features, enabling fully automated and unsupervised brain state classification in epilepsy.

## Introduction

Despite decades of research, current approaches to epilepsy treatment remain unsuccessful in about a third of patients, and the mechanisms of the disease remain insufficiently understood^1^. It has been highlighted how epilepsies and seizures are highly heterogeneous^2,3^ and how treatment responses vary not only across patients but also across time^4,5^. Therefore, the modern approach to epilepsy treatment, as well as medicine in general, aims to tailor interventions to the individual and their current needs in a closed-loop and data-driven manner^6–8^.

Trying to explain (some of) the heterogeneity in epilepsy, current advances in epilepsy research study epilepsy as a dynamic disease^9^. It has been shown how patient-specific fluctuations in seizure likelihood on the scale from hours to days govern seizure emergence^2,10–15^. Moreover, varying levels of seizure likelihood could explain the variability in treatment responses to brain stimulation: The same stimulation, that helped prevent a seizure when delivered at low seizure likelihood, could trigger a seizure at high seizure likelihood^4,5^, a phenomenon that can be explained by fundamental dynamical systems principles^16^. Similarly, the varying efficiency of epilepsy surgery to render a patient seizure-free was linked to the percentage of resected brain areas that, in retrospect, were identified to have the highest seizure likelihood^17–19^. Finally, the variable patterns of seizure transitions could be classified according to a fixed set of “dynamotypes”^20^. The brain dynamics underlying the fluctuations in seizure likelihood can be viewed as a dynamical system, the state of which develops based on some intrinsic laws of motion and is additionally driven by some endogeneous or exogeneous noise or forces^21–24^. While the space of possible brain states is very high-dimensional and continuous, just a subset of possible states corresponds to (at least transitionally) stable and attracting solutions. Attraction and stability in this context refer to the tendency of the brain to remain in or approach those states, such as a ball rolling down a slope and moving slower or coming to rest inside a valley until reaching or being pushed into another valley. The other brain states, such as the ball’s visited positions while rolling down the slope, are unstable and therefore are visited for a negligible amount of time only. Thus, the dynamics may be reasonably simplified by considering the brain state progression through a smaller, discrete set of distinct states (such as “resting state” and “seizure state”) corresponding to the ball positions when the ball has come to or is close to rest.

In the last years, studying recorded brain activity to uncover the latent brain states and the time points of their transitions has gained considerable interest, not just in the field of epilepsy, but also, for example, in the study of sleep and wakefulness^25,26^ and information processing^27,28^. Employed methods typically include clustering analysis of segmented data from functional magnetic resonance imaging (fMRI), scalp, or intracranial electroencephalography (EEG or iEEG). Adding to such data-driven approaches, dynamical systems theory allows to characterize the observed brain states and their transitions (e.g., by linking distinct dynamical patterns to “resting state” and “seizure state” and showing how a change in a model parameter can lead the system to switch between the states). As observable from the recorded time series, the state-specific dynamic patterns can be simulated using models (sets of ordinary differential equations) given an initial condition and, potentially, some realization of dynamical or observation noise. Changing model parameters, different activity patterns, such as resting, oscillating, or spiking behavior, can be reproduced via simulations. Furthermore, transitions between different patterns can be studied, providing mechanistic insight and the means to predict or even control the likelihood of certain transitions^29,30^. Thus, in epilepsy, dynamical systems theory has provided valuable mechanistic insight helping to characterize epileptic brain states and the different mechanisms that could govern seizure emergence^31–34^. Recently, dynamical brain models have further been introduced as tools to predict individualized treatment outcome and test beds for developing treatment strategies in general^8,30,35,36^; see Dallmer-Zerbe et al.^37^ for a recent review. However, to date, no clinically available applications are explicitly contingent on dynamical systems theory or computational modeling. Thus, a bridge between theory and application is yet to be built.

This study aims to propose a model-driven classification of epileptic brain states using a well-known, bio-physiologically realistic model of epilepsy dynamics^38,39^. In contrast to two recent studies^40,41^, that also used a model-driven approach, the proposed classification procedure is built on a prototype comparison using different types of model-generated activity. As in our case, only the model output (prototypical time series) is used; the modeling environment can be easily replaced in the developed pipeline. Furthermore, we include a direct comparison of the model-driven classification strategy with a data-driven one and a comparison between a generalized and individualized classification strategy. Data samples in this study include hippocampal rat slices *in vitro* (high potassium model), as well as intracranial EEG recordings (iEEG) collected in the human hippocampus from patients with temporal lobe epilepsy (TLE).

## Results

### Types of brain state in epilepsy

**Figure 1:**
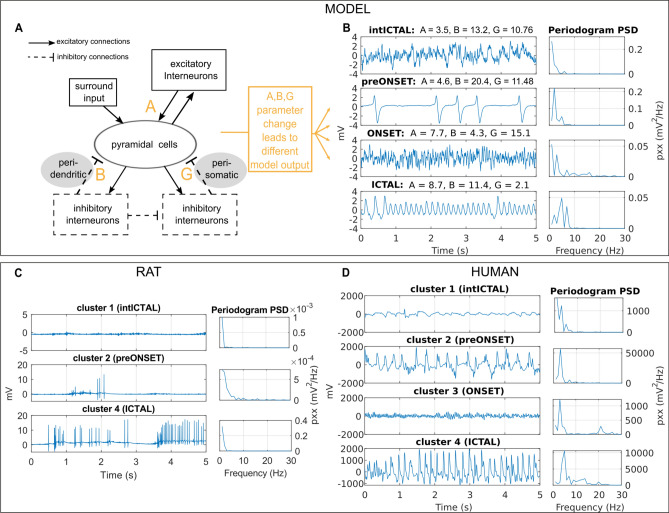
Types of epileptic brain state during epileptic transitions in the model, rat, and human electrophysiology. (**A**) The Wendling model^39^ of neuronal population dynamics with two inhibitory interneuron populations, one communicating with the pyramidal cell population through somatic synapses (fast inhibition) and one through dendritic synapses (slow inhibition). The synaptic gain parameters A, B, and G control the amplitude of postsynaptic potentials of excitation, and slow and fast inhibition, respectively. (**B**) Under different model configurations of A, B, and G, the model output, the summed average postsynaptic potentials on pyramidal cells, resembles electrophysiological recordings during four distinct tissue states. (**C**,**D**) Randomly selected exemplary time series for each one of four clusters in rat 1 and human 1. Clusters are matched with the four types of brain state defined by Wendling so that cluster 1 is the interictal, cluster 2 is the preonset, cluster 3 is the onset, and cluster 4 is the ictal type of brain state (see Methods section). Rat data segments did not include onset type segments due to faster and more local dynamics.

Wendling et al.^39^ describe four types of brain state in epilepsy (interictal, preonset, onset, and ictal) that can be observed from hippocampal recordings from patients with TLE and are reproduced by their computational model. In this study, we simulate the four types of brain state in epilepsy using the Wendling model (Fig. 1A, B) and show that they match the types of brain state identified through clustering segmented data from in vitro rat local field potential (LFP) recordings (high-potassium model of epilepsy, Fig. 1C) and human iEEG recordings (Fig. 1D). The interictal type of brain state is characterized by random fluctuations around a constant mean voltage of recorded activity. The preonset type of brain state features occasional high amplitude spikes, while the onset type has high-frequency oscillations, typically in 15 to 40 Hz frequency bands. Finally, the ictal type of brain state shows ongoing rhythmic activity, typically in the range of 4 to 10 Hz. Note that in the rat LFP recordings (see Fig. 1C), the captured dynamics seemingly take place at a higher temporal scale, and thus there was no observed onset type brain state.

### Automatized brain state classification

**Figure 2:**
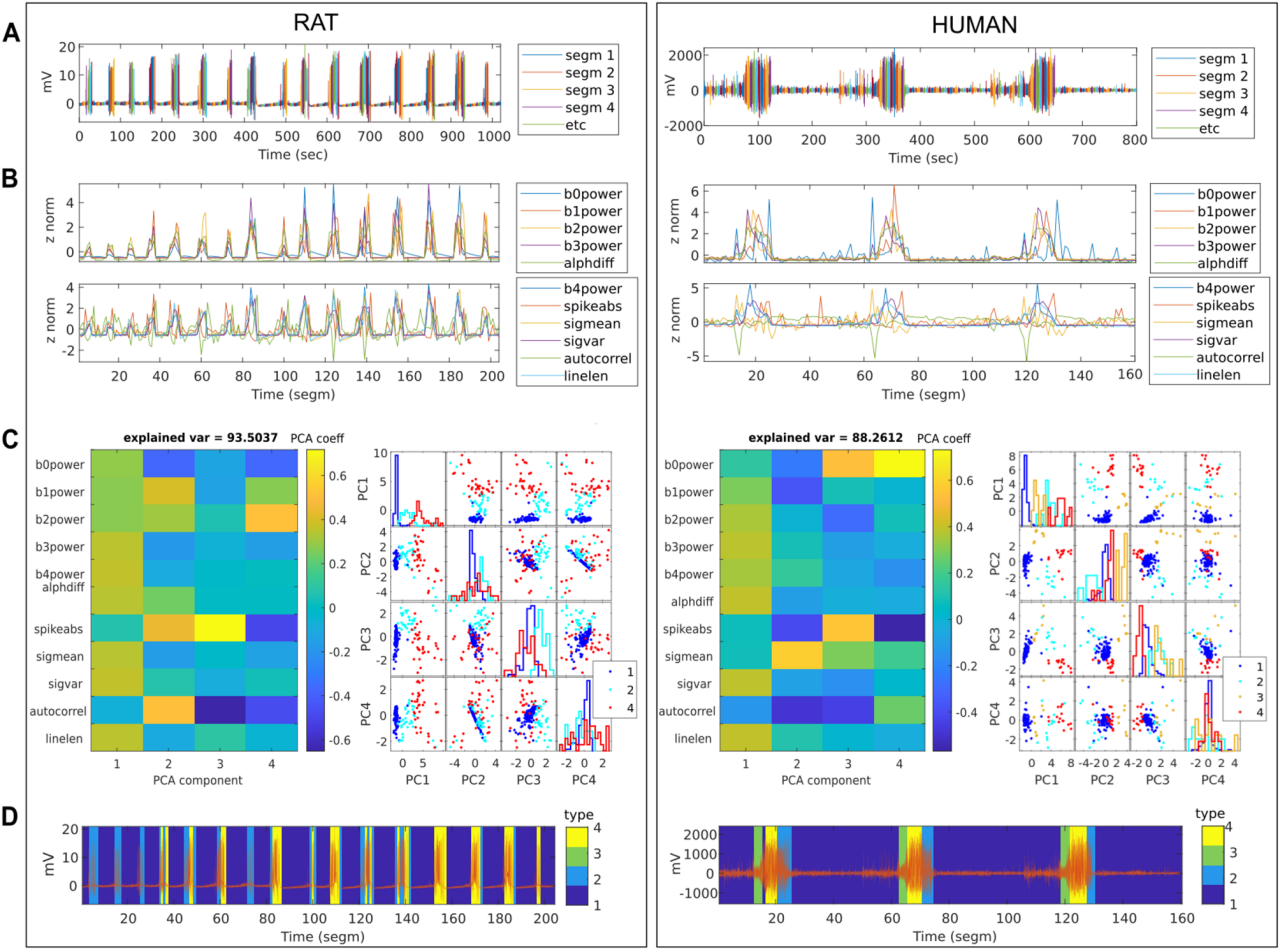
Individualized, data-driven brain state classification in rat 1 (left) and human 1 (right). (**A**) Continuous rat LFP and concatenated human iEEG seizure recordings are segmented into 5-s segments. (**B**) 11 signal features are calculated for each 5-s segment and displayed over time. Characteristic changes in feature values indicate the current brain state concerning seizure dynamics. (**C**) Dimensionality reduction from 11 features to four principle components (PC). Factor loadings for each of the features (left) show a shared contribution to PC 1 and main contributions to PC 2–4 by auto-correlation, spike count (spikeabs), and band power in slower frequencies (b0- to b2power $\le$ 12 Hz). Cluster identity of each data segment (right, color-coded for type 1: interictal, 2: preonset, 3: onset, and 4: ictal) shows well-separated clusters in rat and human data, especially for PC 1. (**D**) Resulting data-driven brain state classification over time. Color code in the background of each data segment indicates which of the cluster centroids from data-driven clustering was closest to the segment’s PCA-projected signal features. The brain state classification shows to identify time points of seizures and their transitions reliably. However, in the human data (right), Wendling-defined preonset type of brain state (type 2, light blue) is often detected during the transitions out of instead of into seizure.

**Figure 3:**
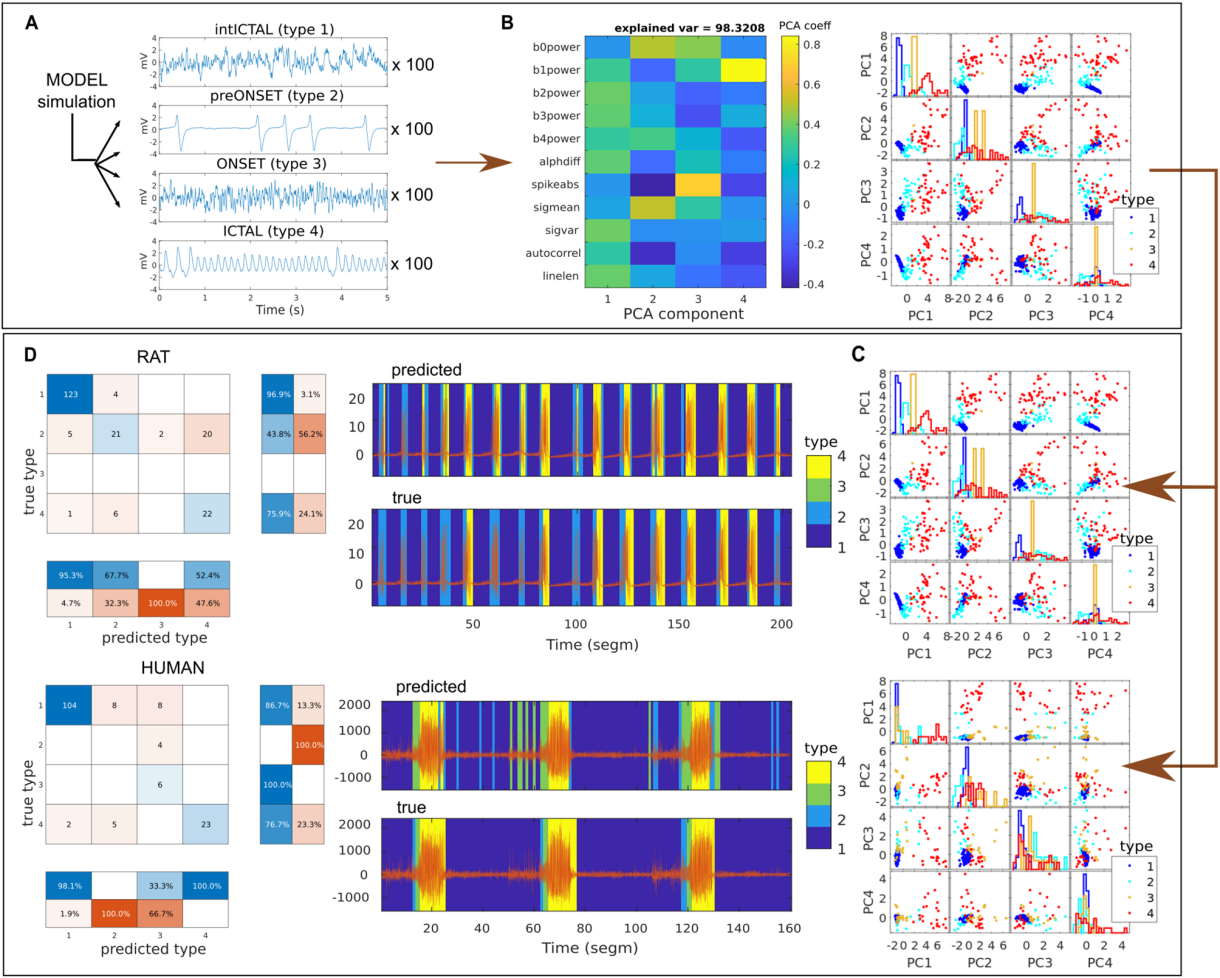
Generalized, model-driven brain state classification of real data segments. (**A**) Model simulation of different types of brain state in epilepsy. Due to noise, the model output varies slightly over the 100 iterations performed for each type of model configuration. (**B**) Cluster analysis on features calculated from the 400 simulated data segments. Feature loadings on the four components show very similar patterns to data-driven clustering (Fig. 2C, left). The four types (right, color-coded) form separated clusters with small overlaps of type 1 and type 2 due to varying amounts of spikes in the preonset segments (type 2). (**C**) Example rat (top) and human data (bottom) are projected into model-driven PC space and assigned a color-coded type label based on the closest model centroid. (**D**) The assigned label (predicted) is then compared to the visually obtained label (true). In the displayed dataset, classification performance (left) was especially good for interictal and ictal types of brain state and preonset type in rats; however, low for preonset type in humans.

The proposed procedure for automatized brain state classification from electrophysiological data is based on the four types of brain states in epilepsy and the comparison of each segment of data to the corresponding epileptic brain state prototypes. The prototypes are generated following five sub-steps (further elaborated below): data segmentation or simulation, feature calculation, principle component analysis, cluster analysis, and centroid labeling. The labeled centroids then serve as prototypes, and individual data segments are labeled according to the label of the most similar prototype, i.e., the closest centroid. Classification performance is assessed against visual labeling for real data segments or against the known type-specific model configuration for simulated data segments. It is assessed in the dataset used for prototype generation to evaluate individualized classification performance (Table 1 and Fig. 2), as well as in all other datasets for assessing the generalizability of the prototypes (Table 2 and Fig. 3). Data- and model-driven classification differ regarding the dataset used for prototype generation: in model-driven classification, the prototypes are generated based on simulated segments with type-specific model configuration (100 segments per type). In data-driven individualized classification, the prototypes are generated based on (all) data segments of a given rat or human. For assessing the generalizability of the data-driven classification, we use the prototypes obtained from the rat and human with the highest number of data segments to classify the data of all others. Notably, even the data-driven classification is model-informed in that the centroid labeling during prototype generation is always based on Wendling-type simulations. This way, the suggested data-driven approach is also wholly unsupervised (i.e., no visual expert assessment of data segments needed) and thus fully automatized while utilizing the mechanistic understanding of epileptic dynamics encoded in the well-established dynamical model.

Individualized, data-driven brain state classification is visually summarized in Fig. 2 for the rat and the human with the most recorded seizures (continuous recording in rat, concatenated in humans), and thus the highest number of data segments, respectively (rat 1 and human 1 in Table 1). After cutting the data into segments of five-second length (Fig. 2A), we calculate 11 signal features expected to indicate the current epileptic brain state. The features are chosen based on literature, where they have proven their utility to (a) differentiate between the four types of brain state in epilepsy in particular^39,42^ or (b) to capture critical slowing during epileptic transitions^4,14^ The features are: the signal mean, the average band power in different frequency bands (b0power: 0–0.5 Hz, b1power: 0.5–4 Hz, b2power: 4–12 Hz, b3power: 12–64 Hz, b4power: >64 Hz), two spike measures (alphdiff: distance between 0.05 and 0.95 quantile; spikeabs: spike count as the number of outliers based on Tukey 1.5 interquartile range threshold), as well as signal variance (sigvar), auto-correlation (autocorrel) at 5 ms lag, and line length (linelen). We find that feature values change over time and seem to be indicative of the current epileptic brain state, such that, for example, autocorrel typically drops at the onset, and b1power and spikeabs usually peak at the end of a seizure (Fig. 2B). Furthermore, it is apparent that the features behave similarly and thus overlap in the information they capture about seizure dynamics.

To eliminate redundant information and thus help differentiate the epileptic brain state types based on the features, principle components analysis (PCA) was conducted, reducing the dimensionality from the 11 features to four principal components. The biggest part of captured information, PC 1, is shared among most features. Additional information is captured by power in lower frequency bands, spikeabs, and autocorrel, which in turn substantially contribute to PC 2–4 (Fig. 2C left panels). In the next sub-steps, the projected signal features (i.e., the PCA scores) for all data segments are clustered into four clusters, and the cluster centroids are matched with the Wendling-defined types (so that, e.g., the centroid of cluster 2 becomes the prototype for type 4: ictal), to obtain the labeled brain state prototypes. Figure 2C right panels shows that PC 1 reliably differentiates interictal (type 1) and ictal (type 4) state, while PC 2 helps to differentiate preonset (type 2) and onset (type 3) state. Moreover, human and rat-driven clustering show astonishing similarity, especially for the interictal state (across PCs) and ictal state (PC 1). Finally, for each data segment (dot in Fig. 2C right panel), we identify the labeled centroid (prototype) that is spatially closest in PC space, thus most similar, to predict the type label of each segment (color-coded in Fig. 2D). Predicted labels are then compared against visual data labels to obtain individualized sensitivity and positive predictive value (PPV) performance.

As Table 1 shows, individualized model-driven brain state classification performs best. In data-driven classification, individualized rat data classification performs better than individualized human data classification. Overall, higher classification performance is achieved in larger datasets, and more of the Wendling types match the clusters of observed brain states. Only in human dataset 1 does data clustering manage to identify all four types of brain states in epilepsy. Testing the mean sensitivity and PPV across datasets of each species for significance, we find that classification results are always highly significant (*p* < 0.01, for group level permutation testing against classification performance for Markov chain simulations, see Methods: Statistical Analysis), with *mean* ± *STD* sensitivity in the model: 0.99, rat: 0.73 ± 0.07, or human: 0.45 ± 0.15, and PPV in the model: 0.99, rat: 0.79 ± 0.16, or human: 0.56 ± 0.10.

**Table 1:** Individualized classification performance for all datasets. .

	Model	Rat 1	Rat 2	Rat 3	Human 1	Human 2	Human 3	Human 4
Sensitivity	**0.99**	0.74	0.79	0.65	0.63	0.51	0.45	0.39
PPV	**0.99**	0.75	0.66	0.97	0.66	0.64	0.79	0.46
# segments	**400**	204	110	108	160	127	98	70
# prototypes	**4**	3	3	2	4	3	2	3

Model-driven classification, here applied to model data only, is marked in boldface. Generally, bigger datasets (higher # segments) had higher sensitivity, and more identified clusters matched the Wendling types (hence higher # types).

### Generalizability of prototypes across datasets in data- and model-driven brain state classification

Using the four prototypes obtained from any dataset, we can also label data segments from other datasets based on the closest prototype, e.g., using rat-driven prototypes to label human data segments. Figure 3 visually summarizes generalized, model-driven brain state classification for rat and human data. After simulating data segments using different model configurations for each type of brain state (Fig.  3A) and calculating the signal features, PCA, clustering, and centroid labeling (Fig.  3B), feature vectors obtained from rat and human data segments can be projected into (and labeled in) the model-driven PC space (Fig. 3C). For performance assessment, the predicted segment label (predicted type) is then again compared against the visually assessed segment labeling (true type; Fig. 3D). For ease of visual comparison with Fig. 2, Fig. 3 displays the same data of the rat and the human with the longest recording (rat 1 and human 1). The same individuals are used for generalized, data-driven brain state classification. Classification performance across all individuals of another (or the same species, excluding the one individual used for prototype generation) for model-, rat- or human-driven prototype generation is shown in Table 2.

**Table 2:** Generalizability of prototypes across different datasets. .

Prototype origin	Model		Rat		Human	
	Sensitivity	PPV	Sensitivity	PPV	Sensitivity	PPV
Model-driven	0.99**	0.99**	0.77** ± 0.05	0.65** ± 0.11	0.56** ± 0.07	0.52** ± 0.05
Rat-driven	0.69**	0.81**	0.76** ± **0.04**	0.70** ± **0.10**	0.50** ± 0.08	0.61** ± 0.07
Human-driven	0.56 *	0.84**	0.62** ± 0.07	0.54** ± 0.09	0.47** ± **0.11**	0.55** ± **0.11**

The mean sensitivity and standard deviation of type-averaged sensitivity and PPV rates are shown across all dataset individuals. In the case of labeling data of a given species (values on diagonal, marked in boldface), the individual whose data were used to derive the brain state prototypes was excluded. Model-driven prototype origin led to the best results for sensitivity in all datasets and had better PPV than human-driven classification. Rat-driven classification had higher PPV in the rat and human datasets. All classifications had performance significantly above the chance level: ** significant with *p* < 0.01; * significant with *p* < 0.05.

We find that model-driven brain state classification is generalizable and thus reliably detects the type of brain state in epilepsy in our rat and human dataset, with highly significant mean sensitivity and PPV rates across the different types of brain state (see Table 2, row ’model-driven’). Also, rat- and human-driven brain state classification (see row ’rat-driven’ and ’human-driven’) performed significantly above chance (*p* < 0.01 in rat and human datasets). While sensitivity was consistently highest for model-driven classification, rat-driven classification had higher PPV in both the rat and the human data set.

Comparing the mean sensitivity and PPV rates in rats and humans between the individualized and generalized classification strategies (Table 2 vs. Table 1), we find that for model-driven classification, the generalized approach achieved higher sensitivity, but lower PPV than the individualized approach (sensitivity: rat 0.77 > 0.73, human 0.56 > 0.45; PPV: rat 0.65 < 0.79, human 0.52 < 0.56). Also, rat- and human-driven classification showed the pattern of higher sensitivity but lower PPV in generalized vs. individualized (rat-driven classification in rat, sensitivity: 0.76 > 0.73, PPV: 0.70 < 0.79; human-driven classification in human, sensitivity: 0.47 > 0.45, PPV: 0.55 < 0.56).

Overall, the generalized, model-driven approach achieved the best mean sensitivity in both the rat and human data set (rat 0.77, human 0.56). The best mean PPV in the rat was achieved by the individualized, data-driven approach and in the human by using generalized rat-driven classification (rat 0.79 and 0.61, respectively).

## Discussion

In this study, we propose epileptic brain state classification for data segments according to four types of brain state in epilepsy as described in Wendling et al.^39^. Notably, the prototypes for each type of brain state can be obtained from simulated, rat, or human data, respectively. Prototype generation included several sub-steps of data analysis, including feature calculation, principle component analysis, clustering, and centroid labeling (crucially, model-informed even during data-driven classification).

We find that the type of brain state of a given data segment was predicted significantly above chance for all datasets and strategies (model-driven vs. data-driven and individualized vs. generalized). Model-driven classification achieved higher sensitivity but lower PPV than data-driven classification, a pattern we commonly observed when comparing the generalized and individualized approaches. However, the model-driven approach not only generalized better to other datasets overall (Table 2) but achieved even higher sensitivity than using the same data to be also classified for prototype generation (individualized classification: Table 1). The classification of model-generated segments was particularly successful in terms of PPV. However, this might be partially ascribed to the homogeneous distribution of simulated segments between the four classes, which generally improves average PPV compared to imbalanced classes. This is a general problem faced in real data, given the varying occurrence rate of the types. As proposed in this study, a model-driven approach has the natural advantage that the number of segments used for prototype generation is up to the experimenter. However, even in the imbalanced rat and human data, the achieved PPV was relatively high and significantly above the random classifier expectation.

The overall results of this study suggest that epilepsy dynamics manifest with consistent electrophysiological patterns across species well-captured by the Wendling model. For epilepsy diagnosis and treatment, this implies that brain state classification (1) is automatizable in an unsupervised and mechanism-enlightening manner, and (2) might not need to be tuned to the individual to achieve good classification results (especially to achieve high sensitivity), despite the substantial heterogeneity among patients. These implications will be discussed in the following, alongside a more detailed discussion of our findings.

### Model-driven brain state classification is unsupervised and provides mechanistic insight

In recent years, significant advances have been achieved in the field of data-driven, automatized seizure detection for epilepsy (dealing with the classification of ictal against other states). Available seizure detection devices for EEG and non-EEG data have proven their efficiency primarily for tonic-clonic seizures^43,44^. Tracking epilepsy brain states over time, including transition states such as the preonset and onset type in this study, is a much more complex problem. Debates about the existence of a preictal brain state, as well as whether or not slow processes (like a successive loss of network resilience^4^) govern seizure emergence, are on-going^45,46^. Due to the mixed outcomes from the countless attempts to characterize and detect the preictal state, the research community has moved on to focus on detecting the so-called pro-ictal state, i.e., the state of increased seizure likelihood. Thus, our focus has shifted from trying to predict seizures deterministically towards seizure forecasting following a probabilistic approach^47^. As it has been shown, computational modeling, replicating different patterns of observed activity and providing means to test the mechanisms of their transitions, can serve as tools to fill in our gaps of understanding and better characterize current brain states^29,33^. We consider this study, the same as the work of Song et al.^40,41^, an essential step on the way to making use of the advantages of modeling for modern brain state tracking for epilepsy, adding to the successes of data-driven strategies, instead of competing with them. Our results highlight that the model-driven approach can add to the data-driven strategy, achieving comparable or even better results than a data-driven approach. We demonstrate that a model can generate a set of “pure” prototypes (further elaborated below), which we believe lies at the heart of modeling: creating a highly simplified yet helpful representation of reality. Our results, in particular, support the usefulness of the four Wendling types of brain state for hippocampal recordings from TLE patients and the high potassium rat *in vitro* model. While the classification performance of real data in this study, overall, did not meet some of the seizure detection performances achieved in the literature, the achieved values in the range of 0.42 to 0.99 can be considered quite successful, given that the expected sensitivity in four-way classification in case of random assignments is 25%. It is important to note that the performance was not homogeneous across the states. As expected, it was typically the highest for the interictal and ictal states and lower for the transition types. This can be explained by the inherently heterogeneous nature of the segments corresponding to the transitions to and back from seizure. Furthermore, different types of seizures are preceded and followed by different types of brain states in epilepsy^20,31,35^. Thus, further studies are needed to extend our results beyond the four types of brain states and the narrow choice of epilepsy type and recording location in this study.

To eliminate redundant information and thus help differentiate the brain state types based on the features, principle components analysis (PCA) was conducted, reducing the dimensionality from the 11 features to four principal components. The biggest part of captured information, PC 1, is shared among most features. Additional information is captured by power in lower frequency bands, spikeabs, and autocorrel, which in turn substantially contribute to PC 2–4 (Fig. 2C left panels). In the next sub-steps, the projected signal features (i.e., the PCA scores) for all data segments are clustered into four clusters, and the cluster centroids are matched with the Wendling-defined types (so that, e.g., the centroid of cluster 2 becomes the prototype for type 4: ictal), to obtain the labeled brain state prototypes. Figure 2C right panels shows that PC 1 reliably differentiates interictal (type 1) and ictal (type 4) state, while PC 2 helps to differentiate preonset (type 2) and onset (type 3) state. Moreover, human and rat-driven clustering show astonishing similarity, especially for the interictal state (across PCs) and ictal state (PC 1). Finally, for each data segment (dot in Figure 2C right panel), we identify the labeled centroid (prototype) that is spatially closest in PC space, thus most similar, to predict the type label of each segment (color-coded in Fig. 2D). Predicted labels are compared against visual data labels for individualized sensitivity and PPV performance. We found the lower sensitivity of the data-driven classification might be a consequence of the limited amount of data segments to derive the prototypes (a problem that data-driven classifiers often face). Real data segments contain noise (produced by other ongoing brain processes, artifacts, and measurement noise) and segments of mixed type (e.g., first half onset, second half ictal type). This is one of the major reasons why data-driven classifiers typically require large, multi-center datasets and an appropriate time scale for data segmentation to identify reliable types of brain states. Data-driven prototypes of brain states that are identified via cluster analysis, thus, may be epileptic or tied to other brain processes taking place at the same time. Therefore, assuming that the modeled types of brain state capture relevant activity patterns of epileptic brain states, clustering model data has, by default, the advantage of providing somewhat “pure” type representatives instead of the more noise- and segmentation bias-prone clustering of real data. Thus, noise and segmentation bias most likely influenced the performance of data-driven classification in this study. That we did not find any onset type segments in the rat data could be an example. The captured dynamics in rat LFP recordings seemingly take place at a higher temporal scale (see Fig. 1). This could be attributed to (a) capturing neuronal activity from a more localized environment, i.e., from a few neurons instead of from thousands as recorded by the human iEEG macro-electrodes and (b) generally faster transitions between interictal and ictal states in the rat slice (see Fig. 2A left panel, approximately one seizure per minute), than in the human intracranial recording (see Fig. 2A right panel, approximately one seizure per three hours; for plotting purposes inter-seizure interval is cut so that only 1 min before until 1 min after the seizure is kept). In the case of (b) segmentation bias might have played a role. Presumably, the 5-s interval for data segmentation was too long to contain mostly the high-frequency type of activity. Also, other choices in the pipeline could have influenced the results: for example, the chosen number of components of PCA kept for further analysis. We chose four components, as they contained more than 80% of variance for all datasets (88% in humans, 94% in rats, and 98% in model data). However, optimal thresholds are data-specific. Furthermore, overall, we worked with a limited sample size. Therefore, real data classification results might not be sufficiently robust. Overall, due to the outlined considerations, the performance of data-driven classification in this study could have been underestimated.

A significant practical advantage of the proposed model-driven approach, when compared to the data-driven approach, is that brain state labeling is achieved unsupervised, i.e., without the need for visually classified data to serve as ground truth for building the classifier. Visual labeling is a lengthy and cumbersome procedure requiring much clinical expertise, especially for classifying transition types of brain states. Thus, in this study, visual labeling was only used to validate the classification results so that even data-driven classification was partially model-informed and thus unsupervised. It is pretty remarkable that each data cluster in the human data (human 1) could be uniquely assigned one of the Wendling types and that in the rat data, where no onset type of brain state was present, the onset label was the only one not assigned to any of the clusters. This finding supports our main conclusion of consistent patterns in brain states in epilepsy across species. Yet, in shorter datasets, there were fewer type matches (Table 1). The different numbers of prototypes might have influenced the classification results. For example, in the rat data without onset type data, there were only three types of brain state to detect and sensitivity, increasing the chance level to assign a correct type label. On the contrary, classification performance was likely to be decreased in datasets where centroids were dropped because the same type label was given to two or more centroids.

Another vital advantage of the model-driven approach mentioned above is that it can add relevant mechanistic insight. In the employed model, the epileptic brain states are explained and linked in the mechanical framework of hippocampal excitation and inhibition levels. This is particularly relevant in the context of brain state tracking for the application of timing-specific intervention. There, defining the current brain state based on a specific balance in excitatory and inhibitory population dynamics (A, B, G parameters in the Wendling model) could have direct implications for the desired treatment effects (e.g., a reduction of peri-dendritic inhibition to achieve a transition from preonset type back to the interictal type of brain state). Classifying brain state according to four prototypes with given model parameters, as done here, is a more conservative approach than the one that was used by Song et al.^40,41^, where the authors fit model parameters to individual data segments and infer brain states based on classifying the identified parameters. Our approach is more conservative in the sense that the specific model to generate the time series of prototypical activity for a given brain state is less relevant and, thus, can be more easily replaced (e.g., to obtain patient-specific prototypes from data-informed phenomenological models such as in^35,36^).

### Model-driven brain state classification might help tackle patient-heterogeneity

The high heterogeneity among and within epilepsy patients and their seizures is one of the main challenges in epilepsy treatment, which has been suggested to be overcome by treatment approaches tailored to the individual patient and time point^6,7^. The proposed brain state classification approach provides a tool to assess the current brain state in a data-driven and, thus, time and patient-specific manner. We assessed the potential advantage of characterizing brain states, i.e., deriving prototypes, in a patient-tailored way over the generalizing approach, i.e., across-subject brain state classification. The individualized brain state classification had better PPV but worse sensitivity than the generalizing approach. Keeping in mind that sensitivity levels for the data-driven strategy might have been underestimated due to the limited sample size in this study and that the occasional smaller number of derived prototypes in the individualized approach might have influenced the results, we speculate that, especially when little data is available for a given patient (and a given type of brain state), the generalized approach might be preferable. The model-driven classification performed best among the different prototype datasets for the generalized approach. Conversely, generalized human-driven classification achieved the lowest performance in this study, suggesting that generalizing from one human to another might be problematic. Therefore, we propose that model-driven classification might better capture generalizable patterns and thus help overcome some of the observed patient heterogeneity in epilepsy. Note that the human dataset of this study was rather homogeneous, as it was recorded in the hippocampus from patients with hippocampal, temporal lobe epilepsy. Therefore, the presented results do not sufficiently address the patient heterogeneity due to the etiological background and varying locations of the epileptic focus. However, the types of brain state and model environment used here have been observed and applied outside of temporal lobe epilepsy^48,49^ Future work is needed to assess the performance of the suggested approach to model-driven classification in bigger and more heterogeneous samples. Due to the chosen prototyping approach, the model and features are easily replaceable in the presented pipeline, which could thus be tailored to different patterns and types of brain states, if needed.

## Conclusion

This study highlights the potential of model-driven brain state classification in epilepsy and provides a pipeline for unsupervised model- and data-driven classification based on prototype comparison. We are convinced that the potential of model-based approaches for clinical application is still on the verge of being explored. Future research for a potential clinical application of model-driven brain state classification includes optimization of the performance of suggested procedures, a possible extension of the types of brain states, validation on a more extensive dataset, and a comparison with other machine learning schemes.

## Methods

### Study design

We compare the performance of epileptic brain state classification across different strategies (generalized classification: model-, human- and rat-driven; individualized classification: every individual for themselves) in three data sets (model, human, rat). The different employed strategies can be understood as treatment conditions. The outcome measures of this study are the sensitivity and PPV rate. They are assessed against the visual expert assessment of the data and statistically tested against the classification performance in 1000 realizations of a matching Markov Chain (permutation testing) per individual, which can be understood as the control condition in this study. Statistical testing was done on a group or individual level for generalized or individualized classification. The visual expert assessment was blinded to the predicted brain state of a given data segment. The experimental unit was a single rat or human. The sample size in this study was not calculated a priori but depended on the data available to us, fulfilling our data-specific selection criteria (see below). Table 1 shows the amount of data segments per individual.

### Data

#### Simulated data

We simulate the Wendling neural mass model^38,39^ of interacting populations in the hippocampus as implemented by Fietkewicz et al.^42^ (customized code available at our GitHub repository), using the parameters listed in Table 3.

**Table 3:** Model parameters used for simulations.

Parameter	a	b	g	C	C1	C2,C7	C3,C4	C5	C6	Sigmoid v0, e0, r	Noise mean, std
Value	100	30	350	135	C	0.8 C	0.25 C	0.3 C	0.1 C	6, 2.5, 0.56	90, 30

Changing additional parameters A, B, and G leads to different signal patterns in the model output representing the EEG signal. We simulate 100 segments of five seconds length at a sampling rate of 512 Hz for each type of brain state in epilepsy, given A, B, and G values from Wendling et al.^39^: interictal: A = 3.5, B = 13.2, G = 10.76; preonset: A = 4.6, B = 20.4, G = 11.48; onset: A = 7.7, B = 4.3, G = 15.1; ictal: A = 8.7, B = 11.4, G = 2.1.

#### Rat data

The data were obtained from three male Wistar rats (approximately 200 g) that underwent the following procedure. Before the experiment, animals were housed in an enriched environment in 12/12 h light and dark conditions. The health status of the animals was checked regularly. For the experiment, the animals were deeply anesthetized (ketamine 80 mg/kg, xylazine 25 mg/kg) and then decapitated. The brains were removed from the skull and placed into ice-cold, oxygenated protective solution sucACSF (containing mMol 189 Sucrose, 2.5 KCl, 0.1 CaCl$_2$, 5 MgCl$_2$, 26 NaHCO$_3$, 1.25 NaH$_2$PO$_4 \cdot$ H$_2$0, and 10 glucose). The brain was cut in the sagittal plane into slices of 350 $\mu$m thickness using a vibratome (Campden Instruments, Loughborough, UK). The hippocampus was cropped, and the CA3 area was cut off. The hippocampal slices were stored, at room temperature, in a holding chamber filled with “normal” ACSF consisting of (in mMol) 125 NaCl, 26 NaHCO$_3$, 3 KCl, 2 CaCl$_2$, 1 MgCl$_2$, 1.25 NaH$_2$PO$_4$, and 10 glucose, aerated with a humidified 95% O2–5% CO$_2$ mixture. After >60 min, slices were transferred to an oxygenated interface recording chamber (34 ± 1$^{\circ }$C), constantly superfused with normal ACSF. Local field potentials were recorded using extracellular glass microelectrodes (diameter 10–15 $\upmu$m) filled with ACSF. Signals were amplified (AC/DC Differential Amplifier Model 3000, A-M Systems, Inc., Carlsborg, Washington, USA) and digitized (Power1401, CED, Cambridge, England) with a sampling frequency of 10 kHz and stored using Spike 2 for further analysis. The slices were left for at least 10 min in the recording chamber to accommodate them. Then, the recording electrode was put on the stratum pyramidale. For spontaneous seizures to emerge, we increased the K+ concentration by adding KCl solution in large steps (1–3 mMol) until a total concentration of 6.5–7 mMol was reached. Then we increased the concentration further in small steps (0.2–0.5 mMol) until spontaneous seizure-like events occurred. The Ethics Committee of The Czech Academy of Sciences approved all experimental procedures. All methods were performed in accordance with the relevant guidelines and regulations, and the study is reported in accordance with ARRIVE (Animal Research: Reporting of In Vivo Experiments) guidelines. The data used in this study were chosen upon the occurrence of spontaneous seizure-like events up until the time point of stimulation, which was later applied to the slices for a different experiment. Recordings of Rat 1 included a total of 16 seizure-like events (Rat 2: 6, Rat 3: 7).

#### Human data

Intracranial EEG data were obtained from four patients recorded during their presurgical workup at Motol Epilepsy Center in Prague, Czech Republic, between 2009 and 2017. Data were recorded using macro depth electrodes (Dixi Medical or Ad-Tech) in the referential montage using Stellate Harmony (sampling frequency 1 kHz) or Natus NicOne (sampling frequency 512 Hz). The study was performed in accordance with the Declaration of Helsinki, ethical approval was granted by the Ethical Committee of Motol University Hospital, and written informed consent was obtained from all subjects. For this study, patients were retrospectively selected based on their type of epilepsy (temporal lobe epilepsy), that they had at least one resected channel in the hippocampus and had excellent surgery outcomes (Engel Ia 2 years after surgery). In those patients, data were selected from each recorded seizure, particularly the time interval ranging from one minute before the start to one minute after the end of the seizure, from a hippocampal and resected channel. iEEG contacts in hippocampal structure CA1 were identified by assigning MNI coordinates to the probabilistic cytoarchitectonic atlas using SPM Anatomy toolbox^50^(*version 2.2c*), and the channel with the highest probability was selected for analysis. Individual patient characteristics are summarized in Table 4.

**Table 4:** Individual patient characteristics, as well as the recording location of the contact selected for this study, and the number of recorded seizures. .

Patient	Gender	Type of epilepsy	Age (epil. duration)	Rec. location (probability)	n seizures
Human 1${}^{\textrm{a}}$	Male	Left temporal	28 (8) years	CA1 (100%)	3
Human 2	Male	Left temporal	54 (28) years	CA1 (92%)	3
Human 3	Female	Left temporal	41 (23) years	CA1 (94%)	2
Human 4	Female	Right temporal	23 (4) years	CA1 (99%)	1

Recording location according to probabilistic cytoarchitectonic atlas (see text for details).

${}^{\textrm{a}}$Human 1 was used for prototype generation in human data-driven brain state classification.

#### Data preprocessing and visual labeling

There was no data preprocessing, apart from centering the rat and human data around zero and inverting their polarity (to achieve consistency between rat, human, and model recordings). Furthermore, data were 50 Hz filtered to exclude line noise. Data were segmented into five-second segments, and for the rat and human data, each segment was assigned to one of the four Wendling types based on a visual assessment that took into account (1) the clinical marks of seizure on- and offset and (2) the order of brain state types (e.g., onset preceded ictal and was preceded by preonset). Figure 3D shows an example of visual assessment results. Visual labeling was not necessary for simulated data; for each segment, the used A, B, and G parameters—and thus the true type of brain state—was known.

### Brain state classification procedure

Brain states are classified based on a prototype comparison procedure. The prototype generation follows these sub-steps: (1) feature calculation, (2) principle component analysis, (3) clustering analysis, and (4) centroid matching to Wendling types. Matched centroids then serve as prototypes, and any data segment can be classified by choosing the most similar of the prototypes. Code will have been made available at our GitHub repository at https://github.com/cobragroup/epileptic-brain-states by the publication date.

#### Feature calculation and normalization

We chose 11 signal features: signal mean, the average band power in range 0–0.5 Hz, 0.5–4 Hz, 4–12 Hz, 12–64 Hz, and >64 Hz, the distance between 0.95 and 0.05 quantile, spike count as the number of outliers based on Tukey 1.5 interquartile range threshold, signal variance, auto-correlation at 5 ms lag and line lengths; due to their demonstrated usefulness to differentiate between the specific four types of brain state in epilepsy considered in this study^39^ and to characterize seizure transitions in general^4,14^. Features are calculated on each data segment separately and then z-normalized across all segments of a given individual or, in the case of the model, across all 400 simulated segments. Normalization is done to account for different scaling of the signal features.

#### Principle component analysis

For prototype generation, we aim to reduce the dimensionality by removing information that was shared among features. Therefore, we conducted the principal component analysis with four components, which always accounted for more than 80% of the variance in all datasets used for prototype generation (88% in humans, 94% in rats, and 98% in model data). To be able to project other data into the same PC space, PCA coefficients need to be stored.

#### Cluster analysis and centroid matching to Wendling types

**Figure 4:**
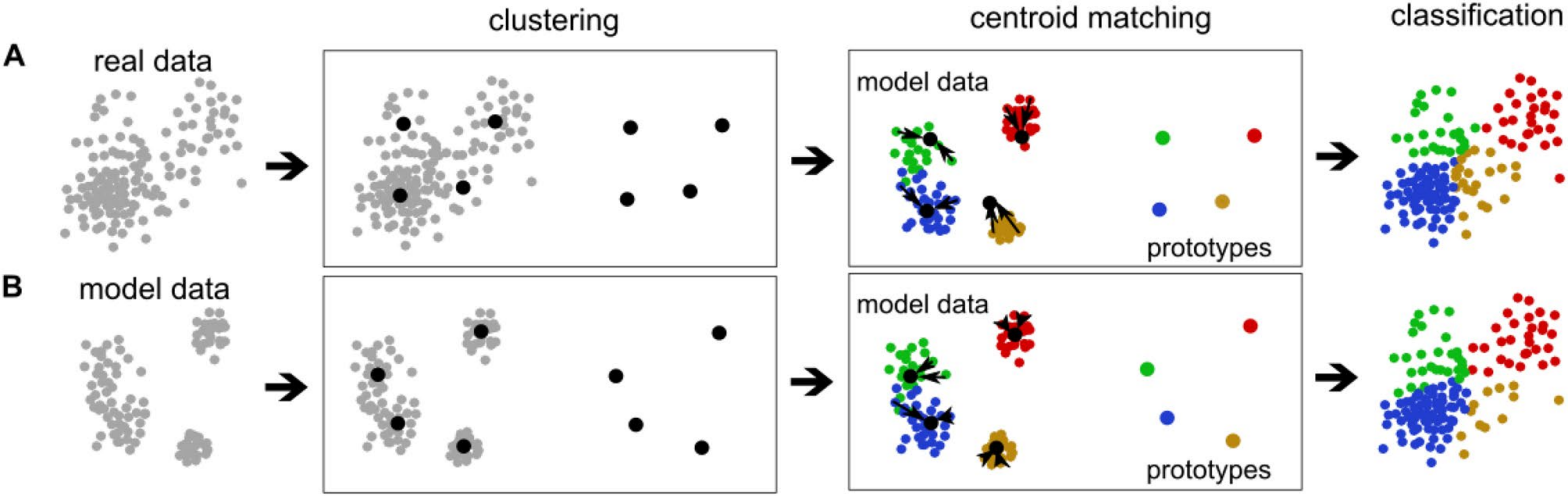
Brain state classification procedure. (A) Data-driven brain state classification. Real data segments (dots) are clustered to obtain centroids (black dots) labeled during centroid matching. Centroid matching is carried out via ’voting’ among all closest model data segments, using their known type of brain state, given the model configuration used to generate them. The most commonly assigned model type becomes the new centroid label, indicated by color: interictal—blue; preonset—green; onset—yellow; ictal - red. Labeled centroids then serve as prototypes, and individual data segments are classified by identifying the closest prototype. In this example, the same dataset used clustering is classified for individualized classification. (**B**) Model-driven brain state classification of real data. Model data segments are clustered and labeled to obtain the model-driven prototypes. In this example, the obtained prototypes are used to classify the real dataset from (**A**), the same as for generalized classification.

In the next step of prototype generation, we cluster the PC data using *kmeans* algorithm to obtain cluster centroids which then serve as the prototypes for brain state classification. We choose four clusters of brain state, as our goal is to find clusters matching the four Wendling types. We then ask which of the four obtained clusters corresponds to which type of brain state (Fig. 4 “centroid matching”). We need to assign each cluster a unique type label to answer this question. For this, we use the model dataset, as in this dataset, the ground truth of the brain state type for each segment is known. Note that this step is done both in the model- and data-driven classification, as it solely matches the data-driven clusters with the Wendling-type brain state. Thus, in data-driven classification (Fig. 4A), real data is used to carry out the dimension reduction, clustering, and defining the prototypes as the centroids of the clusters, however, the model data is used to assign the four interpretable labels to the clusters. Therefore, for each segment of model data, z-normalized signal features are projected into the PC space of the prototype dataset. We then carry out a Voronoi tiling of the PCA space concerning the cluster centroids: to each centroid, we associate the region of all points, i.e., model data segments, that are closer to this centroid than to any of the remaining three centroids. Then the label for a given centroid is decided by voting among the model data segments within each region.

In some individuals, two of the centroids received the same label by this procedure; in this case, we kept only the prototype with the higher number of label-defining simulated segments, while the latter was dropped. Sometimes no model segment fell into the region defined by a centroid, again leading to omitting the centroid. Thus, in some individuals, we find fewer prototypes than four (marked as # types in Table 1).

#### Classification of a new data segment

Using the obtained prototypes (model, rat or human; colored dots in Fig. 4), we classify the type of brain state of any data segment by projecting its z-normalized feature values into the respective prototype dataset-derived PC space by multiplying them with the individual PCA coefficients and identifying the closest prototype centroid. That centroid’s type label (color in Fig. 4) becomes the segment’s label.

### Statistical analysis of classification performance

For statistical analysis, we calculate the confusion matrix for predicted versus true type (see Fig. 3D) for each individual. Then, we calculate sensitivity and positive predictive value (PPV) before averaging across the four types of brain state as:

$$\begin{aligned} { sensitivity} = \frac{{ True Positives}}{{ True Positives} + { False Negatives}} \quad { PPV} = \frac{{ True Positives}}{{ True Positives} + { False Positives}} \end{aligned}$$

Significance testing is done on the group level for each species (model, rat, or human) by comparing the group mean sensitivity or PPV against group mean sensitivity or PPV in 1000 surrogates (discrete-time, finite-state, time-homogeneous Markov chain simulations using the given data-specific true type transition matrix). For the model, the type transition matrix contains equal amounts of transitions for all types. Reported *p*-values correspond to the percentage of data realizations (including all surrogates and the single original data) that achieved the same or a higher sensitivity or PPV than the obtained group mean sensitivity or PPV, respectively.

## Data Availability

The datasets generated and analyzed during the current study are available from https://osf.io/8cdz5/.

## Code availability

All data processing and analysis were conducted in Matlab version *R2021a* (Mathworks Inc., USA). Relevant code is publicly available at the COBRA group GitHub repository at https://github.com/cobragroup/epileptic-brain-states.
